# Glutathione Provides a Source of Cysteine Essential for Intracellular Multiplication of *Francisella tularensis*

**DOI:** 10.1371/journal.ppat.1000284

**Published:** 2009-01-30

**Authors:** Khaled Alkhuder, Karin L. Meibom, Iharilalao Dubail, Marion Dupuis, Alain Charbit

**Affiliations:** 1 Université Paris Descartes, Faculté de Médecine Necker-Enfants Malades, Paris, France; 2 INSERM, U570, Unité de Pathogénie des Infections Systémiques, Paris, France; Stanford University School of Medicine, United States of America

## Abstract

*Francisella tularensis* is a highly infectious bacterium causing the zoonotic disease tularemia. Its ability to multiply and survive in macrophages is critical for its virulence. By screening a bank of *HimarFT* transposon mutants of the *F. tularensis* live vaccine strain (LVS) to isolate intracellular growth-deficient mutants, we selected one mutant in a gene encoding a putative γ-glutamyl transpeptidase (GGT). This gene (*FTL_0766*) was hence designated *ggt*. The mutant strain showed impaired intracellular multiplication and was strongly attenuated for virulence in mice. Here we present evidence that the GGT activity of *F. tularensis* allows utilization of glutathione (GSH, γ-glutamyl-cysteinyl-glycine) and γ-glutamyl-cysteine dipeptide as cysteine sources to ensure intracellular growth. This is the first demonstration of the essential role of a nutrient acquisition system in the intracellular multiplication of *F. tularensis*. GSH is the most abundant source of cysteine in the host cytosol. Thus, the capacity this intracellular bacterial pathogen has evolved to utilize the available GSH, as a source of cysteine in the host cytosol, constitutes a paradigm of bacteria–host adaptation.

## Author Summary

The role of nutrient acquisition systems in survival and multiplication of intracellular bacterial pathogens within infected cells is yet poorly understood. The data presented here suggest that *Francisella tularensis*, a highly infectious facultative intracellular bacterium, is capable of utilizing glutathione (GSH) and γ–glutamyl-cysteine peptides present in the cytosol of infected host cells. An *in vitro* negative selection method, based on the use of a bacteriostatic antibiotic, to recover intracellular growth mutants directly from a pool of mutants, allowed us to select one mutant in a gene encoding a γ-glutamyl transpeptidase (GGT). The mutant strain showed impaired intracellular multiplication and was strongly attenuated for virulence in mice. The cleavage of these cysteine-containing peptides by GGT activity provides thus the essential source of cysteine required for intracellular multiplication. The capacity *F. tularensis* has evolved to utilize GSH, the most abundant source of cysteine in the host cytosol, constitutes a model of bacterial adaptation to intracellular lifestyle.

## Introduction

*Francisella tularensis* is a Gram-negative bacterium capable of causing the disease tularemia in a large number of mammalian species. It is a highly infectious bacterium that can be transmitted to humans in numerous ways. Four different subspecies (subsp.) of *F. tularensis* that differ in virulence and geographic distribution exist. These are designated subsp. *tularensis* (type A), *holarctica* (type B), *novicida* and *mediasiatica*. *F. tularensis* subsp. *tularensis* is the most virulent subspecies causing a severe disease in humans, whereas *F. tularensis* subsp. *holarctica* causes a similar disease but of less severity [1]. Because if its high infectivity and lethality, *F. tularensis* is considered a potential bioterrorism agent [2].

*F. tularensis* is a facultative intracellular bacterium that infects and replicates mainly inside macrophages, but which can also infect hepatocytes, endothelial cells, epithelial cells, fibroblasts, chicken embryos, and amoebae [3]. Although the molecular mechanisms by which *Francisella* adapts to life inside host cells are not well understood, a number of genes that are necessary for *Francisella* to survive inside its host and cause disease are known [4]. These include genes located in the *Francisella* pathogenicity island (FPI) [5],[6],[7],[8],[9],[10],[11], the genes encoding the regulatory proteins MglA, SspA, and PmrA, which regulate expression of the FPI [5],[12],[13] and the genes responsible for lipopolysaccharide (LPS) production [14],[15],[16],[17]. Furthermore, several genome-scale screening approaches have been formerly utilized in *F. tularensis* subsp. *novicida*, *tularensis*, *and holarctica*, to identify additional genes that are important for replication inside macrophages or survival in mice [7],[18],[19],[20],[21].

In the present work, we designed an *in vitro* negative selection method, based on the use of a bacteriostatic antibiotic, to recover intracellular growth mutants directly from a pool of mutants. A similar approach has been reported earlier to select intracellular growth-deficient mutants of *Listeria monocytogenes* [22].

One of the mutants isolated by this procedure (see Table S1) showed a drastic intracellular growth defect. This mutant had an insertion into gene *FTL_0766*, encoding a putative gamma-glutamyl transpeptidase, GGT.

We focus here on the role of this gene in the virulence of *F. tularensis*. GGT's are involved in hydrolysis of γ-glutamyl compounds such as glutathione (GSH). GSH is a non-ribosomal tripeptide (L-γ-L-glutamyl-L-cysteinyl-glycine) present in almost all eukaryotic cells and in some prokaryotes. In eukaryotes, GSH is essential for cell homeostasis and GSH-deficiency has been associated with various severe diseases [23]. In mammalian tissues, GSH is generally found in the cytosol at mM levels (1–10 mM) [24]; and GSH biosynthesis and metabolism proceeds through the γ-glutamyl cycle [25]. In all living organisms, GGT catalyzes the first step in the degradation of GSH, which involves the cleavage and transfer of the γ-glutamyl moiety from GSH to an amino acid acceptor (or hydrolysis to release glutamate) and release of cysteinyl-glycine.

The four subspecies of *F. tularensis* require cysteine for growth; and defined media, containing cysteine and other amino acids essential to support growth of *F. tularensis* strains, have been developed [26],[27]. *In silico* analysis of the *F. tularensis* SCHU S4 genome suggests that the specific requirement for cysteine is due to a nonfunctional pathway for sulfate assimilation, resulting from a pseudogene encoding adenylylsulfate kinase [27].

We demonstrate here that *FTL_0766* of *F. tularensis* encodes a genuine GGT involved in the metabolism of γ-glutamyl-containing peptides. GGT allows the utilization of γ-glutamyl peptides as a source of cysteine during intracellular multiplication of the pathogen, and is thus critical for virulence.

## Results

### Selection of *F. tularensis* mutants unable to multiply in J774 macrophages

In the present work, we adapted an *in vitro* negative screening procedure, based on a penicillin selection, for the isolation of *F. tularensis* mutants defective in their ability to replicate intracellularly. *F. tularensis* has been shown to be sensitive to a series of antibiotics [28]. Although penicillins are generally not very active on *F. tularensis*, cephalosporins are more active. In particular, cefotaxime, a third generation cephalosporin with bacteriostatic activity, was shown to be active on *F. tularensis* [28]. This antibiotic was furthermore demonstrated to enter eukaryotic cells [29]. Therefore, since β-lactam antibiotics such as cefotaxime, kill only growing bacteria, a protocol was designed to isolate intracellular growth mutants from banks of transposon insertion mutants.

Banks of mutants were generated in the *F. tularensis* Live Vaccine Strain (LVS), using the *in vivo* Himar strategy (*HimarFT*) [30]. The thermosensitive plasmid pFNLTP16 H3 (which carries a kanamycine resistance cassette between the inverted repeats) was introduced into LVS by electroporation. Transformants were first selected for inheritance and maintenance of the plasmid at permissive temperature (30°C). Then, cultures were shifted at non-permissive temperature (39°C) to inhibit subsequent plasmid replication, and in the presence of kanamycin to select chromosomal integration of the resistance cassette (see Materials and Methods).

Wild-type LVS multiplies in murine macrophage-like cells J774 with an intracellular doubling time of *ca.* 6 h. However, the presence of 1.5 mg ml^−1^ cefotaxime in the culture medium efficiently inhibited multiplication of intracellular LVS (Figure S1). We infected J774 cells with pools of mutant bacteria at an MOI of 100 bacteria/cell in the presence of 1.5 mg ml^−1^ cefotaxime. The surviving bacteria were recovered at selected intervals after infection by plating onto chocolate agar plates (Text S1; Figure S1). Clones isolated by this procedure were tested by Southern blot to select single insertion mutants (Figure S2). Nucleotide sequence analysis of 30 single transposon insertions led to the identification of 10 distinct chromosomal regions (Table S1, Figure S2).

The fact that the largest numbers of mutant hits (10/30) were found in the *ggt* gene (*FTL_0766*), and that the mutation led to the most severe intracellular growth defects (see below), prompted us to focus on this mutant. This gene encodes a putative γ-glutamyl transpeptidase (GGT), a protein of 601 amino acid residues whose sequence is highly conserved among the *Francisella* subspecies (99.2% identity with the subsp. *novicida* ortholog FTN_1159; and 98.8% with the subsp. *tularensis* SCHU S4 ortholog FTT1181c); and significant degree of homology with GGT of *Helicobacter pylori* (47% identity).

### Intracellular growth and virulence of the *ggt* mutant

**Figure 1:**
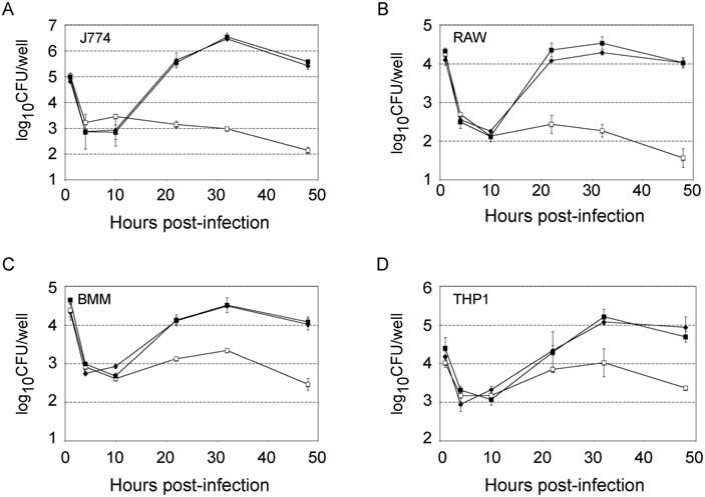
Intracellular replication of LVS, *ggt*, and *ggt-*complemented strains. Replication of LVS, *ggt*, and *ggt-*complemented strains, inside J774 murine macrophage-like cells (A); RAW macrophages (B); mouse bone marrow-derived macrophages (BMM) from BALB/c mice (C); and THP1 human macrophages (D). Results are shown as the average log_10_ CFU/well±standard deviation. Strains are denoted as follows: LVS-pKK214 (◆), *ggt* -pKK214 (□), and *ggt*-pKK-ggt (■).

Growth of the *ggt* mutant was undistinguishable from that of wild-type LVS in broth (Mueller-Hinton, not shown or Chemical Defined medium, see below). The kinetics of intracellular multiplication of the *ggt* mutant was first followed in mouse macrophages over a 48 h-period. The mutant showed a drastic growth defect, with more than a 100-fold reduction of intracellular bacteria in macrophage-like J774 cells after 24 h (Figure 1A). A severe intracellular growth defect of the *ggt* mutant was also observed in the RAW macrophagic cell line as well as in murine bone marrow-derived macrophages (BMM) (Figure 1B and 1C). We then followed intracellular survival of the *ggt* mutant in human macrophages. The mutant showed a severe growth defect in THP1 macrophages, with more than a 10-fold reduction of intracellular bacteria cells after 24 h, and a down to a 20-fold reduction after 48 h (Figure 1D).

**Figure 2:**
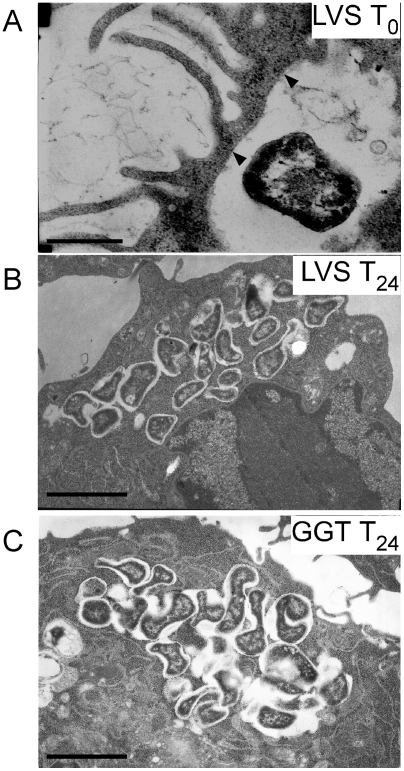
Intracellular electron microscopy. Transmission electron micrographs of thin sections of RAW macrophages infected by LVS or *ggt*, at T0 and after 24 h infection. At T0, *F. tularensis* initially resides in a spacious vacuole at the periphery of the cell (A): LVS. The arrows point to regions where the phagosomal membrane surrounding the bacteria is visible. At 24 h, *F. tularensis* LVS (B) or *ggt* (C) localized in the cytoplasm are surrounded by an electron-lucent space. (B,C) Illustrate cytosolic multiplication of LVS and the *ggt* mutant, respectively. The bar to the bottom left corresponds to 0.2 µm (A) or 1 µm (B,C).

Infection of RAW cells was followed by thin section electron microscopy (Figure 2). As expected, a significant bacterial replication was observed in the cytosol of infected cells 24 h post-infection with wild-type LVS. Of 363 cells counted, 33 were infected by bacteria (9.1%) and 64% of the infected cells contained more than 10 bacteria. Bacterial multiplication had also occurred at 24 h in cells infected with the *ggt* mutant. However, only 5% of the cells were infected by bacteria (34/651 cells) and only 20% of the infected cells contained more than 10 bacteria. These data strongly suggest that the intracellular growth defect of the *ggt* mutant is due to an impaired cytosolic growth.

**Figure 3:**
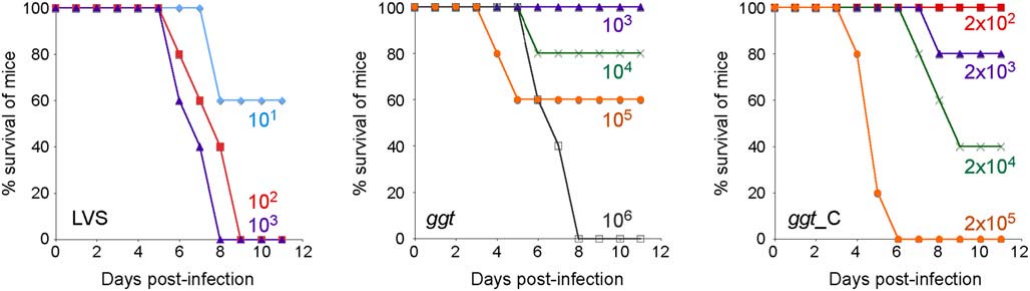
GGT is important for virulence in mice. Survival of BALB/c mice after i.p. infection with LVS, *ggt*, or *ggt*-complemented (*ggt*_C) bacteria. Groups of five mice were infected with the number of bacteria (log_10_) shown next to the curves and followed for 9 days.

The impact of *ggt* inactivation on bacterial virulence was then evaluated by infecting 6–8 weeks old BALB/c mice with the *ggt* mutant and LVS, by the intraperitoneal route (Figure 3 and Table S1). The median lethal dose was determined to be ∼10^1.04^ for LVS and ∼10^4.8^ for *ggt* using the Probit method [31], that is a >1,000-fold attenuation of virulence.

### Functional characterization of the *ggt* mutant

We confirmed that the intracellular growth defect observed in the *ggt* mutant strain was due to the specific inactivation of *FTL_0766* by performing a functional complementation of the gene. For this, the recombinant plasmid pKK-ggt, carrying the wild-type *ggt* allele was introduced into the *ggt* mutant strain. As shown in Figure 1A, 1B, and 1C, the kinetics of intracellular multiplication of the complemented strain, monitored in J774, RAW, bone marrow macrophages, and THP1 cells over a 48 h period, was indistinguishable from that of wild-type LVS harboring the plasmid without insert. In mice, complementation was also observed but appeared to be only partial, possibly to due *in vivo* instability of the complementing plasmid (Figure 3).

GGT activity was determined by measuring cleavage and transfer of a γ-glutamyl moiety, with a standard assay used to monitor blood GGT (performed at the Hospital Necker facility). We monitored γ-glutamyl transpeptidase activity in whole cell lysates of LVS and of the *ggt* mutant strain. Inactivation of *ggt* gene resulted in very low GGT activity (1.9±0.6 U/l) whereas the activity detected in wild-type LVS was of 40.75±0.25 U/l. This shows that *FTL_0766* encodes a γ-glutamyl transpeptidase and that no other protein with γ-glutamyl transpeptidase activity exists in *F. tularensis* LVS. No GGT activity was detected in culture supernatants of LVS and mutant strains, indicating that, under the conditions we tested, the enzyme is not secreted in *F. tularensis*.

The existence of a capsule-like structure surrounding *F. tularensis* has been evoked in several early descriptive papers [32] but its nature was never characterized neither biochemically nor genetically. Interestingly, a study which evaluated the serum resistance of designated “Capsule-positive” and “Capsule-negative” mutants of *F. tularensis*, concluded that LPS is the major part of the capsule and that serum resistance of the Capsule-positive strains is due to the presence of O-side chains of LPS [33]. Moreover, a series of recent studies [14],[16],[34] demonstrated a direct link between LPS O-antigen biogenesis and serum-resistance. In further support of this notion, a very recent paper studied complement-mediated lysis and complement C3 deposition on the *F. tularensis* surface using a series of LVS strains with altered in surface carbohydrate structures, including previously designated Cap- mutants. This study showed that *F. tularensis* resistance to complement-mediated lysis was essentially mediated by the O Ag [35].

**Figure 4:**
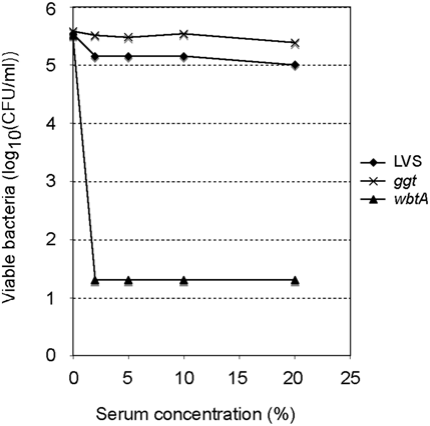
Killing of LVS and mutants by non-immune human serum. Bacteria were incubated with increasing concentrations of serum, and samples were collected after one hour incubation at 37°C. The number of viable bacteria was determined by plating onto chocolate agar plates.

Presence of homologs of the genes *capB* and *capC*, required for capsule biosynthesis in *Bacillus anthracis*, in the genome of *F. tularensis* SCHU S4 [27] have led to the hypothesis that *F. tularensis* might contain a poly-D-glutamic acid (PGA) capsule. Notably, *F. tularensis* mutants in *capB*, and *capC* homologs have been recently isolated in two *in vivo* screening approaches [19],[20]. While these two studies demonstrated the role of these genes in bacterial pathogenesis, they did not provide any information on the actual function of these genes. In *B. anthracis*, *capD* allows the covalent linkage of PGA to the multilayered peptidoglycan and prevents antibodies from gaining access to the bacterium [36]. We have recently reported that *F. tularensis* does not produce poly-D-glutamate in broth [16]. However, since GGT of LVS shares 35% similarity with *B. anthracis* CapD, we examined the impact of *ggt* inactivation on sensitivity to non-decomplemented human serum, using a mutant of LVS devoid of LPS O-antigen and highly sensitive to serum killing (*wbtA*) as a control [16]. The serum had no effect on the viability of the *ggt* mutant and the wild-type strain at all tested concentrations (0–20%), while *wbtA* mutant was efficiently killed at serum concentrations higher than 2% (Figure 4 and Text S1). Hence, we can say that *ggt* is not involved in *F. tularensis* serum-resistance.

### Utilization of extracellular gluthathione and γ–glutamyl-cysteine as cysteine sources

**Figure 5:**
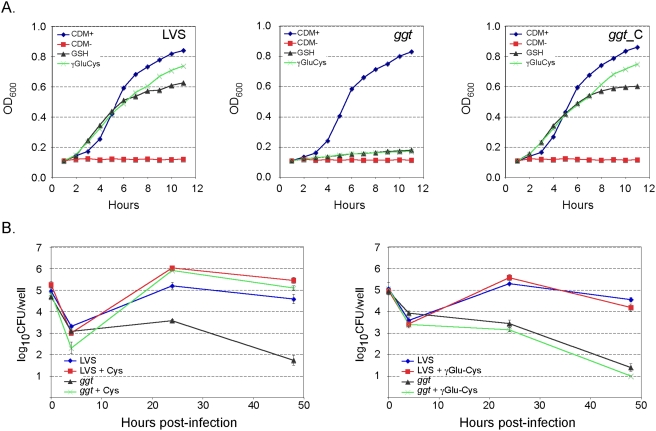
Cysteine is required for *F. tularensis* growth. (A) Growth of LVS and the *ggt* mutant in chemically defined medium (CMD) supplemented with various sources of cysteine. The OD_600_ of the cultures were monitored during 12 h of growth at 37°C with agitation in complete CDM (CDM+) or in CDM lacking cysteine (CDM−) and supplemented with either γ-Glu-Cys or GSH. (B) Replication of LVS and *gg*t mutant inside J774 cells in the presence or absence of cysteine 5 mM (left panel); or in the presence or absence of γ-Glu-cys 1 mM (right panel).

We next examined if the activity of GGT provides a mean for *Francisella* to utilize γ-glutamyl peptides as a source of amino acids and specifically cysteine, which is an essential nutrient for *Francisella* [37]. For this, LVS, *ggt* mutant, and the *ggt* mutant complemented with the plasmid-encoded *ggt* gene were cultivated in defined minimal medium (Chamberlain medium, CDM+) [26] or in CDM lacking cysteine (CDM-). The medium devoid of cysteine was supplemented with cysteine, γ-glutamyl-cysteine (γ-Glu-Cys), or GSH at 100 µM final concentrations. Cultures were incubated at 37°C and growth was monitored over a 12 h-period (Figure 5A). The three strains grew normally in CDM+ medium while no growth was observed in CDM devoid of cysteine (CDM−), confirming the absolute cysteine requirement for growth. Notably, in the absence of cysteine, the addition of glutathione (GSH) or of γ-Glu-Cys partially restored growth of LVS (Figure 5A), indicating that wild-type LVS is able to use these exogenous compounds as a source of cysteine. In contrast, the *ggt* mutant failed to use these compounds (Figure 5A), confirming that the GGT activity encoded by *FTL_0766* is the only enzyme capable of hydrolyzing the γ-glutamyl-cysteine bond of these molecules. As expected, the *ggt* mutant containing pKK-ggt was able to utilize the γ-glutamyl-substates as the LVS strain. The γ-Glu-Cys dipeptide promoted a slightly better growth of LVS than GSH. This observation is compatible with the fact that with GSH as a substrate, the action of an additional peptidase is required to provide cysteine (to cleave the cysteinyl-glycine bond of the dipeptide released after the digestion with GGT).

Notably, *E. coli*, which can utilize exogenous glutathione as a cysteine source in a GGT-dependent manner [38], possesses three cysteineglycinases (the aminopeptidases PepA, B and N), responsible for the subsequent cleavage of the dipeptide cysteinylglycine [39]. We identified the three orthologs of these aminopeptidases in the LVS genome (FTL_1479, FTL_1956, and FTL_1108, sharing 48%, 48% and 38% amino acid identity with PepA, B and N, respectively). It is thus reasonable to assume that LVS has the capacity to efficiently cleave cysteinylglycine peptides to produce cysteine and glycine.

### Functional complementation of *E. coli ggt* by *F. tularensis ggt*

**Figure 6:**
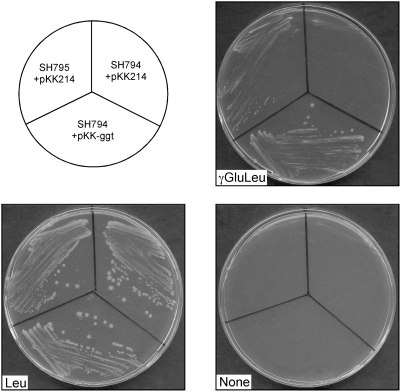
Functional complementation of *E. coli ggt* with *ggt* of *F. tularensis*. *E. coli* auxotrophic strains SH794 (*thr leu ggt*) and SH795 (*thr leu*) containing pKK214 or pKK-ggt (encoding *F. tularensis* GGT), grown on M9 minimal agar plates supplemented with threonine and either leucine (Leu), γ-glytamyl-leucine (γGluLeu), or no leucine source (none).

To evaluate the capacity of *F. tularensis* GGT to functionally complement an *E. coli* GGT-deficient strain, the LVS *ggt* gene (carried on plasmid pKK-ggt) was introduced into *E. coli* auxotrophic strains SH795 (*thr leu*) and SH794 (*thr leu ggt*) [38] (kindly provided by Dr. H. Suzuki, Kyoto Institute of Technology, Japan). Colonies of the *E. coli* strains containing the recombinant plasmid were picked from LB plates and streaked onto M9 minimal agar plates supplemented with threonine and either leucine (M9+Thr+Leu) or γ-glutamyl-leucine (M9+Thr+γ-Glu-Leu). As shown in Figure 6, SH794(pKK-ggt), but not SH794(pKK214) could grow on (M9+Thr+Leu) as well as on (M9+Thr+γ-Glu-Leu). This assay strongly suggests that *F. tularensis* GGT was able to cleave the γ-Glu-Leu bond and, thus, to provide leucine to supplement the leucine auxothrophy of the strain.

### Cysteine is required for intracellular multiplication of the *ggt* mutant

As the *ggt* mutant is unable to utilize γ-glutamyl substrates *in vitro*, we reasoned that the inability of the mutant to replicate inside macrophages results from this deficiency. We speculated that cellular GSH (or other γ-glutamyl peptides) that cannot be used by the *ggt* mutant strain, serve as a source of intracellular cysteine for *F. tularensis*. To test this hypothesis, we cultivated J774 macrophages in DMEM supplemented with 5 mM cysteine and infected them with LVS or the *ggt* strain (non-supplemented DMEM does not contain cysteine but cystine at a 0.22 mM concentration). Addition of cysteine to the cell culture medium promotes increased intracellular growth of the LVS strain compared to growth in J774 cells cultivated in traditional medium (Figure 5B), indicating that cysteine is limiting in the host cytoplasm. When the *ggt* strain was used for infection, bacteria did not multiply in cells cultivated in normal medium (as seen previously, Figure 1A) but multiplied intracellularly in J774 cells cultivated in cysteine-enriched medium (Figure 5B, left panel). In contrast, infection of J774 cells cultivated in the presence of γ-glutamyl-cysteine did not promote growth in the mutant strain (Figure 5B, right panel). The results show that the growth defect of the *ggt* mutant can be circumvented by addition of cysteine to the medium, strongly suggesting that the natural intracellular source(s) of cysteine is a γ-glutamyl-cysteine dipeptide and the abundant tripeptide GSH.

The fact that glutathione is involved in many cellular processes, including oxidative stress defense [40], prompted us to monitor bacterial growth in the presence or absence of 1 mM H_2_O_2_. Growth of LVS and of the *ggt* mutant were unaffected by the presence of 1 mM H_2_O_2_ (see Figure S3), indicating that this concentration of H_2_O_2_ is non-toxic to LVS and that the *ggt* mutation does not increase H_2_O_2_ sensitivity. Of note, this value is still 100-fold above the physiological concentration of H_2_O_2_ that bacteria encounter in phagocytes during the oxidative burst generated by NADPH oxidase, which is in the 1–10 µM range [41],[42],[43],[44]. Thus, it reasonable to assume that that the phenotype of the mutant not related to increased susceptibility to oxidative stress.

### GGT-dependent incorporation of cysteine from GSH into proteins

**Figure 7:**
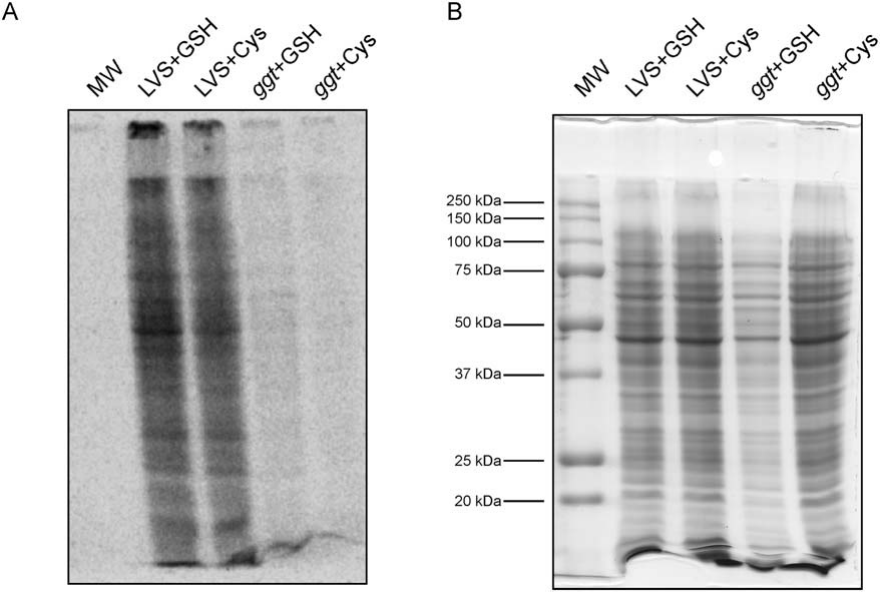
Metabolic labeling. LVS and *ggt* mutant bacteria were grown in CDM devoid of cysteine, supplemented with either GSH (lanes LVS+GSH, ggt+GSH) or cysteine (lanes LVS+Cys, ggt+Cys). Radiolabeled ^35^S-GSH was added to each culture and the suspensions were incubated for 8 h at 37°C with agitation. Bacterial pellets were resuspended in SDS-PAGE loading buffer, and 5 µl of each fraction were loaded onto 10%-SDS-polyacrilamide gels. Each well corresponds to *ca.* 1.8×10^8^ bacteria for LVS+GSH, LVS+Cys, and ggt+Cys; and to 0.6×10^8^ bacteria for ggt+GSH. After electrophoresis, gels were vacuum-dried and scanned with a Molecular Dynamics Phosphorimager. The autogradiograph shown in (A) corresponds to 12 h exposure. As control, a second gel loaded with the same fractions was Coomassie-blue-stained (B).

To further demonstrate that cysteine from GSH was indeed incorporated into proteins, we then performed a metabolic labeling assay (see Materials and Methods for details). Briefly, LVS and *ggt* mutant bacteria were grown in Chamberlain medium devoid of cysteine (CMD−) and supplemented with either 100 µM GSH (LVS+GSH, ggt+GSH) or 100 µM cysteine (LVS+cys, Δggt+cys). ^35^S Radiolabeled-GSH (Perkin Elmers) was added to each culture and the suspensions were incubated for 8 h at 37°C with agitation (in the radiolabled γ-Glu-Cys-Gly molecule, the isotope is on the sulfur of the central cysteine residue CH_2_SH side chain). Bacteria collected by centrifugation were resuspended in SDS-PAGE loading buffer, boiled and loaded onto 10%-SDS-polyacrylamide gels. After electrophoresis, gels were vacuum-dried and scanned with a Molecular Dynamics Phosphorimager. The autogradiograph shown in Figure 7A corresponds to 12 h exposure. As a control, a second gel loaded with the same extracts was Coomassie-blue-stained (Figure 7B). As expected, in CMD− supplemented with GSH, only residual growth was observed with the *gg*t mutant. In contrast, growth of LVS and the *ggt* mutant were identical in CMD− supplemented with cysteine. Radiolabeld protein bands were detected (Figure 7A) only in the two LVS protein extracts (+GSH or +Cys). This assay demonstrates that GSH utilization specifically requires the action of the *ggt* gene product and that cysteine from GSH is directly incorporated into proteins during bacterial growth.

## Discussion

The role of nutrient acquisition systems in survival and multiplication of intracellular bacterial pathogens within infected cells is poorly understood. Using a cefotaxime-based negative selection, we identified a gene, encoding a γ-glutamyl transpeptidase (GGT, *FTL_0766*) that is absolutely required for intracellular multiplication and virulence of *F. tularensis* LVS. The data presented here suggest that *F. tularensis* is capable of utilizing glutathione (GSH) and γ-glutamyl-cysteine peptides present in the cytosol of infected host cells. The cleavage of these cysteine-containing peptides by GGT activity thus provides the essential source of cysteine required for intracellular multiplication.

### Critical role of GGT in *Francisella* pathogenesis

Screenings of banks of mutants have been previously reported in *F. tularensis* [7],[18],[19],[20],[21],[45]. A number of genes were repeatedly found by these different approaches, in particular genes involved in purine/pyrimidine synthesis. Notably, three of these studies identified the *ggt* gene [19],[21],[45] but its properties were not further characterized.

GGT shares some sequence similarity with the CapD enzyme responsible for covalent anchoring of the *B. anthracis* capsule. The existence of a capsule surrounding *F. tularensis* has been evoked repeatedly but never persuasively demonstrated experimentally. Therefore, we evaluated the impact of *ggt* inactivation on serum resistance of LVS as the putative *Francisella* capsule confers serum resistance [46]. The *ggt* mutant showed a serum resistance comparable to that of wild-type LVS, demonstrating that GGT is not involved in serum resistance.

Inactivation of *ggt* had a drastic effect on the ability of LVS to multiply in eukaryotic cells. Strikingly, we showed that the intracellular growth defect of the *ggt* mutant could be eliminated by providing external cysteine to the culture medium, establishing a direct link between GGT activity and cysteine availability. Also, the capacity of the *ggt* mutant to cause disease in mice was severely decreased, further demonstrating the critical role of GGT in pathogenesis.

### GGT allows utilization of γ-glutamyl peptides as a source of cysteine

The involvement of GGT in the utilization of glutathione or other γ-L-glutamyl peptides has been demonstrated in both prokaryotes and eukaryotes [38],[47],[48],[49],[50]. While some bacterial species have the capacity to directly import GSH from the growth medium and degrade it into glutamate and cysteine in their cytoplasm [51], species such as *H. pylori* cannot transport this substrate and must secrete or export GGT to degrade GSH extracellularly. GGT of *H. pylori* has been described as a virulence factor essential for the establishment of the infection in the mouse model [52],[53],[54] and as an apoptosis-inducing protein [55]. Furthermore, a role for GGT in the metabolism of glutamine and glutathione has been recently established, providing information on the role of the enzyme in the physiopathology of this extracellular bacterial pathogen [56]. We did not detect any GGT activity in culture supernatants of LVS under the conditions we tested, suggesting that GGT-mediated degradation of γ-glutamyl compounds occurs in the bacterial cytoplasm. Future work will be required to address the molecular mechanisms of uptake of these compounds in *F. tularensis*.

Our results show that both GSH and γ-glutamyl-cysteine can functionally replace cysteine to support growth of *Francisella* in broth, and that GGT activity is required for this, by cleaving the γ-glutamyl-cysteine linkage. We also showed that *F. tularensis* GGT could functionally complement an *E. coli* GGT-deficient strain. The *F. tularensis* GGT was able to cleave the γ-Glu bond of the dipeptide γ-Glu-Leu, allowing, like the genuine *E. coli* enzyme, the utilization of the dipeptide as a source of amino acid (providing leucine to supplement the leucine auxothrophy of the strain).

Finally, a metabolic labeling assay confirmed that GSH utilization, as a source of cysteine, specifically requires the action of the *ggt* gene product. Furthermore, the use of radiolabeled-GSH, carrying the ^35^S isotope on the cysteine side chain, demonstrated that cysteine from GSH is indeed incorporated into bacterial proteins during growth.

### A model of GSH utilization by intracellular *F. tularensis*

The infectious cycle of *F. tularensis* is essentially intracellular. Since the severe intracellular growth defect of the *ggt* mutant could be completely relieved by the addition of free cysteine to the cell culture medium, it seems most likely that GGT provides a source of cysteine to cytosolic bacteria by degrading internalized γ-glutamyl-cysteine peptides.

GSH is the most abundant non-protein thiol in mammalian cells and the prevalent low-molecular weight peptide in eukaryotic cells, attaining concentrations of 1 to 10 mM in many different cell types, most of which (85–90%) is present in the cytosol. Biosynthesis of GSH is dependent on the availability of the amino acid precursors glutamate, glycine, and cysteine. The intracellular pool of cysteine is relatively small (0.10–0.25 mM) and cysteine is generally the limiting amino acid for GSH synthesis (the other two precursors, glycine and glutamate, are found in considerable higher intracellular concentration). It is estimated that at the low levels of γ-glutamyl-cysteine normally present, 95% is converted to GSH (see for reviews [23],[57],[58] and references therein).

**Figure 8:**
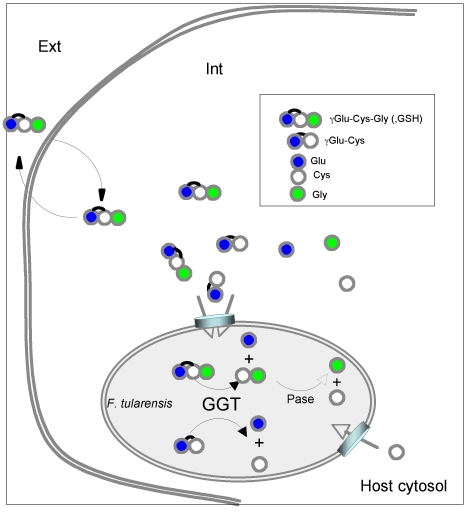
A model of utilization of GSH and γ-Glu-Cys by cytosolic *F. tularensis*. *F. tularensis* requires cysteine for growth. It can utilize GSH (and its oxidized form GSSG), γ-Glu-Cys, cysteine, and cystine as sources of cysteine. In eukaryotic host cells, the intracellular concentration of GSH depends on the availability and transport of cystine (oxidized form of cysteine) and cysteine. Cytosolic GSH, the most abundant thiol-containing compound (present in the mM range), and γ-Glu-Cys serve as sources of cysteine for intracellular growth of *F. tularensis*. In wild-type bacteria, GSH and γ-Glu-Cys are processed by GGT to produce γ-Glu and Cys-Gly or γ-Glu and Cys, respectively. The Cys-Gly dipeptide is further processed by other amino-acid peptidases to produce free cysteine and glycine. GGT-negative bacteria are unable to process these compounds, and the available concentration of free cysteine is too low to promote bacterial growth.

We propose a model for intracellular cysteine acquisition by *F. tularensis* (Figure 8), in which intracellular *F. tularensis* uses γ-glutamyl-cysteine dipeptide and GSH as the primary sources of cysteine. The *ggt* mutant, unable to cleave the γ-glutamyl-cysteine bond, does not obtain sufficient free cysteine (or cystine) from its surroundings to support growth. Therefore, only the addition of mM amounts of free cysteine compensates its enzymatic deficiency. Utilization of cytosolic GSH and/or cysteine-containing dipeptides as a source of cysteine might be used by other intracellular bacterial pathogens.

More generally, acquisition of nutrients should be considered as an integral part of the pathogenicity of intracellular bacteria. This concept is comforted by recent transcriptomic analyses, which revealed the upregulation of many bacterial metabolic and transport functions in infected cells (see for examples [59],[60],[61],[62]). A remarkable example of the interplay between intracellular pathogens and their host has been provided with *L. monocytogenes*. Indeed, it has been shown that a listerial membrane transporter, Hpt, was essential for acquisition of phosphorylated hexoses within the cystosol of infected cells and was, thus, of major importance for bacterial replication [63],[64]. Clearly, intracellular bacterial pathogens have adapted a fine balance between their metabolic needs and the nutrients available (nature and concentration), to multiply efficiently and to avoid a premature death of the host cell that would lead to an aborted infection.

## Materials and Methods

### Strains, media, and chemicals

*F. tularensis* LVS strain was obtained from Dr Anders Sjöstedt (Umeå University, Sweden), and grown at 37°C in Schaedler K3 (Biomerieux SA Marcy l'Etoile, France) broth, Chamberlain defined synthetic medium [26] or Chocolate agar enriched with PolyViteX (chocolate agar) (Biomerieux SA Marcy l'Etoile, France). *E. coli* was grown in Luria-Bertani (LB) medium at 37°C. When required, medium was supplemented with kanamycin (10 µg ml^−1^). Kanamycin was purchased from Sigma-Aldrich (St Louis, MO). Oligonucleotide primers were synthesized by Eurogenetec. Primers and bacterial strains used in this study are listed in Table S2.

### Generation of banks of LVS mutants and *in vitro* screening procedure

Banks of transposon insertion mutants were generated in LVS, by using the *in vivo* Himar strategy (*HimarFT*) [30]. The thermo-sensitive plasmid pFNLTP16 H3 used (kindly provided by Dr. Dara W. Frank, Medical College of Wisconsin), carries, between the inverted repeats, the *npt* gene (determining kanamycin resistance) under the control of the *F. tularensis* LVS *groEL* promoter. Classically, 10 ml of Schaedler K3 medium was inoculated with LVS transformed with Himar 3, and grown overnight at 30°C with shaking to the until late exponential phase (optical density at 600 nm, 0.7–0.9). Cultures were then grown twice overnight at 39°C and once at 40°C in the presence of kanamycin, to eliminate non-integrated plasmid in these bacteria. Cultures were finally stored in 1.5 ml fractions at −80°C.

The murine macrophage-like cells J774 were infected with pools of ca. 10^7^ mutant bacteria (at an MOI of 100 bacteria/cell). After 1 h infection, cells were washed three times with fresh culture medium and incubated for 24 h in DMEM supplemented with 5% (v/v) fetal calf serum, 10 µg m1^−1^ gentamycin (to kill extra-cellular bacteria) and 1.5 mg ml^−1^ cefotaxime (to kill growing intra-cellular bacteria). At given time-points, cells were washed and lysed with 1 ml of sterile water and plated on chocolate agar enriched with PolyViteX. Plates were incubated at 37°C for 3 days and clones obtained were isolated and stocked. Classically, after 24 h of infection, each selection (*i.e.* one 12-wells plate seeded with 10^5^ cells per well) led to the subsequent isolation of approximately 20 colonies on chocolate agar plates containing kanamycin.

#### Screening procedure

Ninety-five clones were isolated after cefotaxime selection and were tested by Southern blot. In 65 clones, multiple bands were detected (from 2 to 5), indicating that several transposition events had occurred (most likely due to non-even loss of the plasmid after passages at non-permissive temperature, as confirmed by a recent study) [21]. These clones were not further studied.

Single insertions were observed in 30 clones (Figure S2). Nucleotide sequence analysis of these 30 single transposon insertions led to the identification of 10 distinct chromosomal regions (Table S1). One double insertion mutant was also analyzed. The genes identified are involved in various processes including metabolism and cell division. Notably, in many instances (Table S1), the same insertion was isolated several times (up to 10 times), possibly due to a bias in the selection procedure after repeated passages at non-permissive temperature and/or during intracellular survival in the presence of cefotaxime.

### Cell cultures and macrophage infection

Macrophage infections were performed as described previously [65] (see Text S1). For determination of numbers of intracellular bacteria, cells were washed three times with fresh culture medium and lysed with 1 ml of sterile water, serially diluted, and plated on chocolate agar.

### Functional complementation

The plasmid used for complementation of the *ggt* mutant, pKK-ggt, was constructed by amplifying a 2,206 bp fragment (corresponding to the sequence 300 bp upstream of the *ggt* gene to 100 bp downstream the gene) using primers D1 and D2 (see Table S2), followed by digestion with *Xba*I and cloning into the *Xba*I site of plasmid pKK214 [66]. The plasmids pKK214 and pKK-ggt were introduced into LVS and the *ggt* mutant by electroporation.

### Functional complementation of *E. coli ggt* by *F. tularensis ggt*

We used the same *E. coli - F. tularensis* shuttle plasmids to evaluate the capacity of *F. tularensis* GGT to functionally complement an *E. coli* GGT-deficient strain. The plasmids pKK214 and pKK-ggt were introduced into *E. coli* auxotrophic strains SH795 (*thr leu*) and SH794 (*thr leu ggt*) [38] (kindly provided by Dr. H. Suzuki, Kyoto Institute of Technology, Japan). Colonies of the *E. coli* strains containing the recombinant plasmid were picked from LB plates and streaked onto M9 minimal agar plates supplemented with thiamine (2 µg/ml), threonine (100 µg/ml), and when indicated either leucine (100 µg/ml) or γ-glutamyl-leucine (100 µg/ml), and incubated for 72 hours at 37°C.

### Metabolic labeling

LVS and *ggt* mutant bacteria were collected from Chocolate agar plates and resuspended in Chamberlain medium devoid of cysteine to a final OD_600_ of 0.11. Each 10 ml culture was supplemented with either GSH at 100 µM final (LVS+GSH, ggt+GSH) or cysteine at 100 µM final (LVS+cys, ggt+cys). Radiolabeled glutathione (^35^S-GSH, Perkin Elmer) was then added to each culture (10 µCi of ^35^S-GSH at a specific activity of 30 Ci/mmol per 10 ml culture) and the suspensions were incubated for 8 h at 37°C with agitation. Bacteria were finally collected by centrifugation and the pellets were resuspended in SDS-PAGE loading buffer. Five 5 µl of each fraction (corresponding to 0.15 ml of bacterial culture) were loaded onto 10%-SDS-polyacrilamide gels.

The culture of LVS+GSH and the two cultures in the presence of cysteine (LVS+Cys, ggt+Cys) grew to a final OD of 0.6, while that of ggt+GSH reached only an OD_600_ of 0.2. Thus, for LVS+GSH, LVS+Cys and ggt+Cys, each well corresponds to *ca.* 1.8×10^8^ bacteria; while for Δggt+GSH, it corresponds only to 0.6×10^8^ bacteria.

After electrophoresis, gels were vacuum-dried and scanned with a Molecular Dynamics Phosphorimager. The autogradiograph shown corresponds to 12 h exposure.

As control, a second gel loaded with the same fractions was Coomassie-blue-stained.

### Gammaglutamyl transpeptidase enzyme assays

Ten ml overnight culture of wild type and the *ggt* mutant were centrifuged and pellets were suspended in 1 ml of Schaedler K3. 0.5 ml of Zorkonium bead was added and mixture was agitated in 3 series of 30 sec at speed of 40 in FastPrep apparatus (GMI, Inc., Minnesota, USA). Samples were centrifuged (3,500×g, 5 min) and supernatants collected (whole cell lysates). GGT activity assay was realized on 50 µl of each sample (corresponding to 10^9^ bacteria), using a standard dosage procedure (Hospital Necker-Enfants Malades, on a Roche/Hitachi analyzer). Values are indicated in U/l, as calculated by the analyser. Four independent measurements were performed.

### Animal studies

Groups of five 6–8 week old female BALB/c mice (Janvier, Le Genest St Isle, France), were i.p. injected with 0.2 ml of *Francisella* in 0.15 M NaCl. The number of viable bacteria used for infection was determined by plating serial dilutions on chocolate agar. The survival of mice was followed for 10 days. Animal experiments were performed according to the INSERM guidelines for laboratory animals husbandry.

## Supporting Information

Figure S1 Cefotaxime screening in J774 macrophages.
(0.05 MB DOC)

Figure S2 Southern blot analysis.
(0.15 MB DOC)

Figure S3 Sensitivity to H_2_O_2_.
(0.04 MB DOC)

Table S1 Mutants selected in the in vitro selection screen and characteristics of mutants.
(0.09 MB DOC)

Table S2 Primers, bacterial strains and plasmids used in study.
(0.12 MB DOC)

Text S1 Loci identified, Intracellular growth and virulence of the *F. tularensis* mutants, and supplementary materials and methods.
(0.06 MB DOC)
