# Supplementary material for: Glutathione Provides a Source of Cysteine Essential for Intracellular Multiplication of Francisella tularensis
Source: PLoS Pathog. 2009 Jan 30;5(1):e1000284. doi: 10.1371/journal.ppat.1000284 (PMC2629122; doi:10.1371/journal.ppat.1000284)
Supplement: Figure S1 — Cefotaxime screening in J774 macrophages. (0.05 MB DOC) [file ppat.1000284.s001.doc]

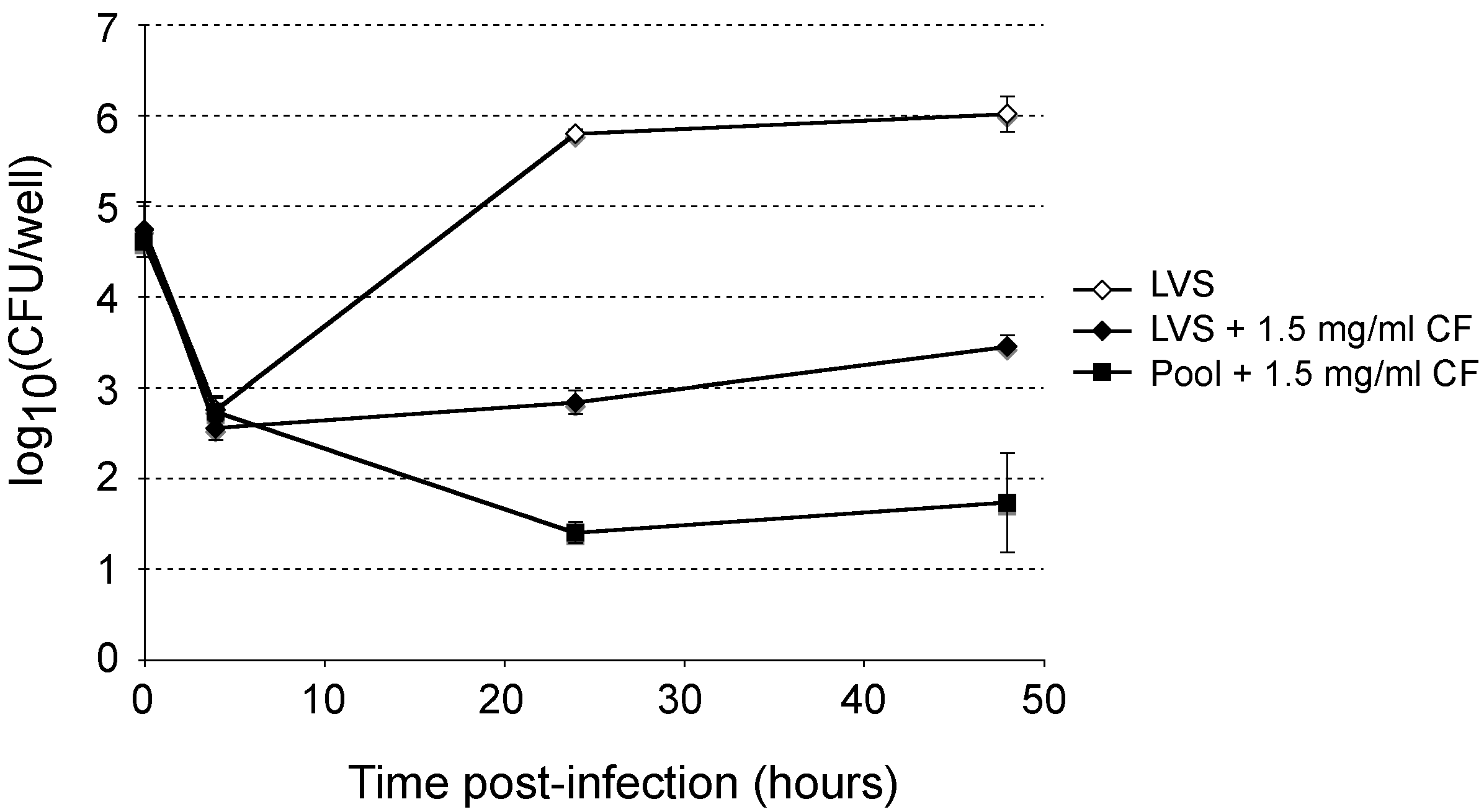


**Figure S1. Cefotaxime screening in J774 macrophages.** J774 cells were infected with pools of *ca.* 10^7^ mutant bacteria. After 1h infection, cells were washed and incubated for 24h with 5% *(vlv)* fetal calf serum, 10 µg m1^-1^ of gentamycin and 1.5 mg ml^-1^ of cefotaxime. At selected intervals, cells were washed and lysed with 1 ml of sterile water. The kinetics of bacterial survival was followed by plating onto chocolate agar plates.
