# Supplementary material for: Glutathione Provides a Source of Cysteine Essential for Intracellular Multiplication of Francisella tularensis
Source: PLoS Pathog. 2009 Jan 30;5(1):e1000284. doi: 10.1371/journal.ppat.1000284 (PMC2629122; doi:10.1371/journal.ppat.1000284)
Supplement: Figure S2 — Southern blot analysis. (0.15 MB DOC) [file ppat.1000284.s002.doc]

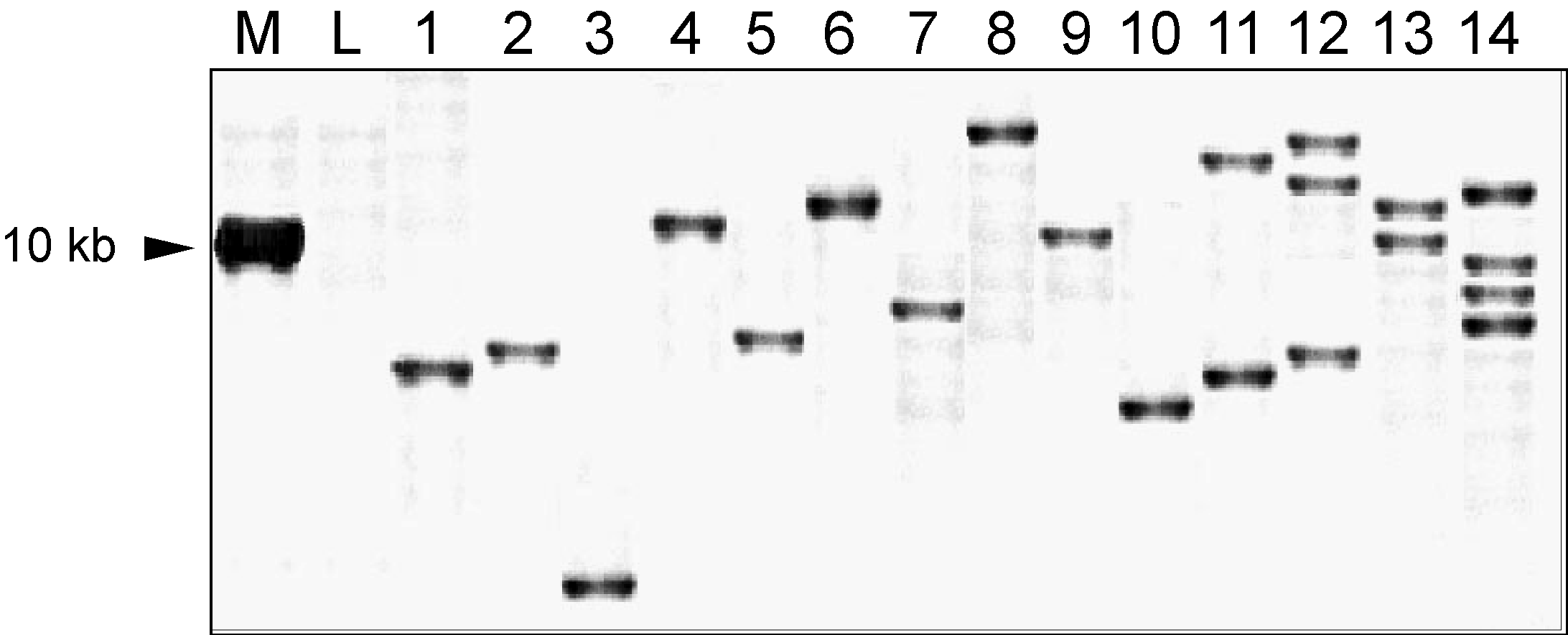


**Figure S2. Southern blot analysis.** Genomic DNAs were digested with *Spe*I overnight, resolved on a 0.7% TAE-agarose gel and transferred to Hybond N+ nylon membranes. Hybridization with a 634-bp, randomly labeled, *npt* probe resulted in single bands of various sizes in the selected clones (lanes 1-10). In a number of clones, multiple bands were detected (illustrated in lanes 11-14). M corresponds to the *Sma*I-linearized plasmid pFNLTP16 H3 ; L, to LVS chromosomal DNA.
