# Supplementary material for: Glutathione Provides a Source of Cysteine Essential for Intracellular Multiplication of Francisella tularensis
Source: PLoS Pathog. 2009 Jan 30;5(1):e1000284. doi: 10.1371/journal.ppat.1000284 (PMC2629122; doi:10.1371/journal.ppat.1000284)
Supplement: Figure S3 — Sensitivity to H2O2. (0.04 MB DOC) [file ppat.1000284.s003.doc]

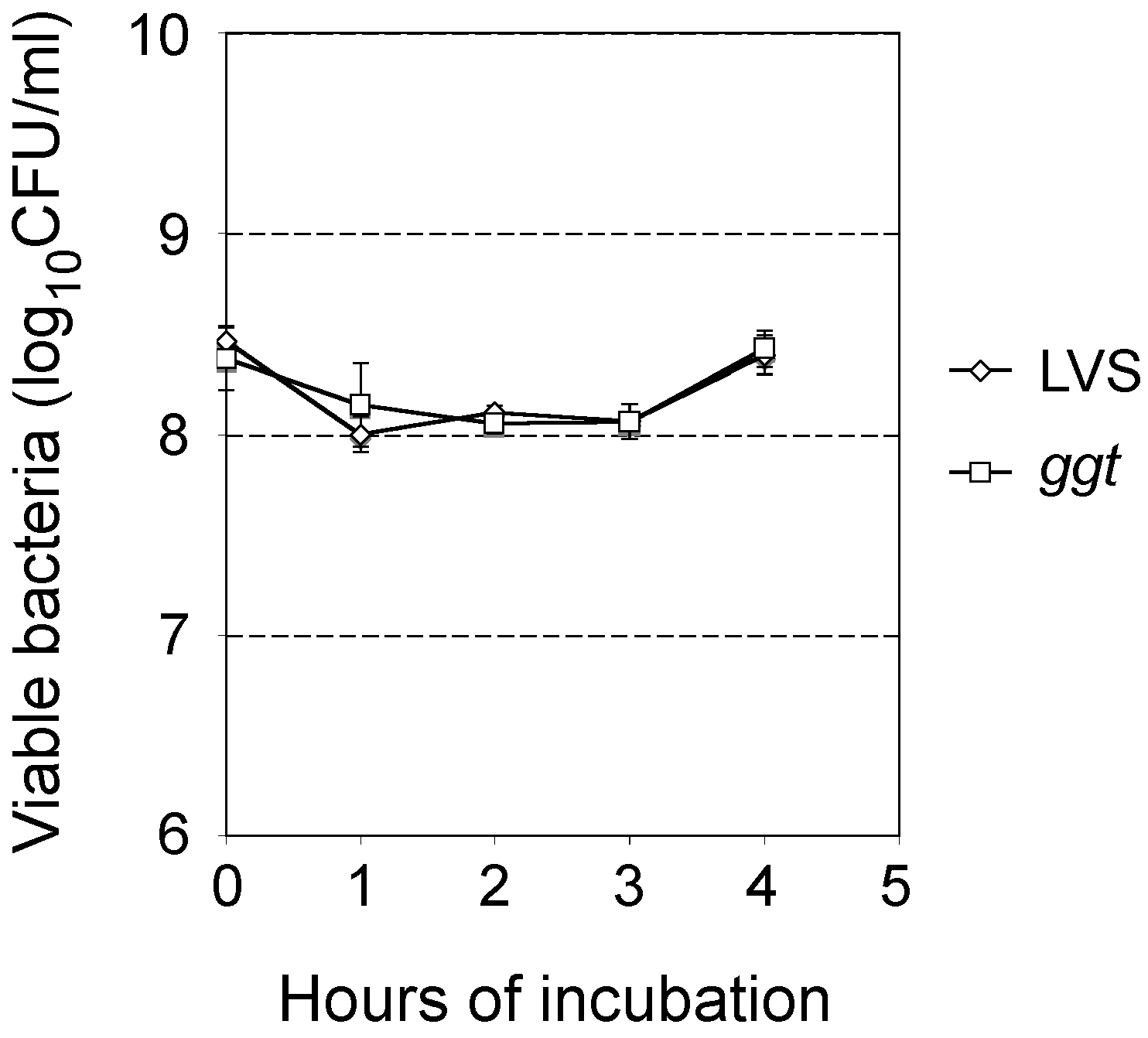


**Figure S3. Sensitivity to H_2_O_2_.** Overnight culture of LVS and *ggt* mutant were diluted in Schaedler K3 (1:10) and 50μl aliquot was removed for bacterial enumeration. H_2_O_2_ was added (to a final concentration of 1 mM) to a one of two bacterial cultures for each strain and one culture was used as control. Cultures were incubated at 37°C for 4h and an aliquot of 100μl was removed every hour to determine the viable bacterial number by plating serial dilution on chocolate agar.
