# Supplementary material for: Glutathione Provides a Source of Cysteine Essential for Intracellular Multiplication of Francisella tularensis
Source: PLoS Pathog. 2009 Jan 30;5(1):e1000284. doi: 10.1371/journal.ppat.1000284 (PMC2629122; doi:10.1371/journal.ppat.1000284)
Supplement: Table S1 — Mutants selected in the in vitro selection screen and characteristics of mutants. (0.09 MB DOC) [file ppat.1000284.s004.doc]

**Table S1:** Mutants selected in the in vitro selection screen and characteristics of mutants

|  |  |  |  | Multiplication in macrophages in vitro^†^ | |  |
| --- | --- | --- | --- | --- | --- | --- |
| Gene | # of isolates | Gene name | Function | J774 | BMM | LD50^‡^ |
| LVS wild-type |  |  |  | +++ | +++ | 10^1.04^ |
| FTL_0766 | 10 | *ggt* | gammaglutamyl transpeptidase | - | - | 10^4.8^ |
| FTL_1240 | 7 | *aroG* | phospho-2-dehydro-3-deoxyheptonate aldolase | + | + | 10^7.2^ |
| FTL_0116/1162 | 1 | *pdpC* | Pathogenity determinant protein | ++ | nd | 10^2.6^ |
| FTL_1724 | 1 |  | Conserved hypothetical protein | ++ | nd | 10^3.1^ |
| FTL_1116 | 1 | *repA* | ATP dependent DNA helicase | ++ | nd | 10^1.1^ |
| FTL_0148 | 2 |  | Sodum/hydrogen exchanger (antiporter) | ++ | nd | 10^1.6^ |
| FTL_0861 | 2 | *ifstu2* | transposase | ++ | nd | nd |
| upFTL_1009* | 1 |  | NA | ++ | nd | nd |
| FTL_0096 | 1 |  | pseudogene | +++ | nd | nd |
| upFTL_1037* | 2 |  | NA | +++ | nd | nd |
| upFTL_1519* | 2 |  | NA | +++ | nd | nd |

^†^ multiplication is scored as follows: +++ (as wild-type LVS), ++ (intermediate), + (low), - (none)

^‡^ determined by Probit method (7).

* up in front of FTL number designates an insertion into the region upstream of the gene

NA: Not Applicable

nd: not determined
