# Supplementary material for: Glutathione Provides a Source of Cysteine Essential for Intracellular Multiplication of Francisella tularensis
Source: PLoS Pathog. 2009 Jan 30;5(1):e1000284. doi: 10.1371/journal.ppat.1000284 (PMC2629122; doi:10.1371/journal.ppat.1000284)
Supplement: Table S2 — Primers, bacterial strains and plasmids used in study. (0.12 MB DOC) [file ppat.1000284.s005.doc]

**Table S2:** **Primers, bacterial strains and plasmids used in study**

| **Primer, strain, or plasmid** | | **Sequence or description** | **Utilization or reference** |
| --- | --- | --- | --- |
| Primer | |  |  |
|  | A1 | 5’-ATTTCCGTGTCGCCCTTATTC | Forward primer β-lactamase (*bla*) probe |
|  | A2 | 5’-TTATCCGCCTCCATCCAGTC | Reverse primer β-lactamase (*bla*) probe |
|  | B1 | 5’-GCTATTCGGCTATGACTG | Forward primer for *HimarFT* (*npt*) probe |
|  | B2 | 5’-CAGCAATATCACGGGTAG | Reverse primer for *HimarFT* (*npt*) probe |
|  | C1 | 5’-GCTTCCTCGTGCTTTACGG | *npt* primer for *HimarFT* insertion sequencing |
|  | C2 | 5’-TGCCACCTAAATTGTAAGCG | R6K primer for *HimarFT* insertion sequencing |
|  | D1* | 5’-CTCTAGAGCGTAATGCCTACATAC | Forward primer for complementation |
|  | D2* | 5’-CTCTAGATAAATTCAGAGAATCATGGG | Reverse primer for complementation |
| Strain | |  |  |
|  | *F. tularensis* LVS | *F. tularensis* subsp. *holarctica* live vaccine strain | A. Sjöstedt |
|  | LVS *ggt::HimarFT* | LVS with a *HimarFT* insertion within FTL_0766 | This study |
|  | LVS *aroG::HimarFT* | LVS with a *HimarFT* insertion within FTL1240 | This study |
|  | LVS *pdpC::HimarFT* | LVS with a *HimarFT* insertion within FTL_0116 or 1162 | This study |
|  | LVS *1724::HimarFT* | LVS with a *HimarFT* insertion within FTL_1724 | This study |
|  | LVS *repA::HimarFT* | LVS with a *HimarFT* insertion within FTL_1116 | This study |
|  | LVS *0861::HimarFT* | LVS with a *HimarFT* insertion within FTL_0861 | This study |
|  | LVS M_IGR1 | LVS with a *HimarFT* insertion upstream of FTL_1009 | This study |
|  | LVS 0096*::HimarFT* | LVS with a *HimarFT* insertion within FTL_0096 | This study |
|  | LVS M_IGR2 | LVS with a *HimarFT* insertion upstream of FTL_1037 | This study |
|  | LVS M_IGR3 | LVS with a *HimarFT* insertion upstream of FTL_1519 | This study |
|  | *E. coli* DH5α | F^-^ φ80*lacZ* Δ*M15 endA1 recA1 hsdR17 supE44 thi-1 gyrA96 relA1 (lacZYA-argF)U169* | Strain collection |
|  | *E. coli* DH5α λ*pir* | DH5α lysogenized with λ*pir* phage | Strain collection, used for *HimarFT* rescue |
| Plasmid | |  |  |
|  | pFNLTP16 H3 | pUC ori, Ft ori (temperature-sensitive), *HimarFT* (*Himar1* with Ft promoter upstream of *npt*), Km^r^, Ap^r^ | (5) |
|  | pKK214 | Ft ori, p15A ori, Tc^r^, Cm^r^ | (3) |
|  | pKK-ggt | *ggt* gene incl. 300 bp upstream and 100 bp downstream cloned in *Xba*I site of pKK214 | This work |

* *Xba*I site is underlined
