# Supplementary material for: Glutathione Provides a Source of Cysteine Essential for Intracellular Multiplication of Francisella tularensis
Source: PLoS Pathog. 2009 Jan 30;5(1):e1000284. doi: 10.1371/journal.ppat.1000284 (PMC2629122; doi:10.1371/journal.ppat.1000284)
Supplement: Text S1 — Loci identified, Intracellular growth and virulence of the F. tularensis mutants, and supplementary materials and methods. (0.06 MB DOC) [file ppat.1000284.s006.doc]

**Text S1**

***Loci identified.*** Ten mutants corresponded to the same transposon insertion into gene *FTL_0766*, encoding a putative gammaglutamyl transferase, a protein of 601 amino acids, hence designated GGT. Seven mutants had an insertion into gene *FTL_1240*. This gene, also designated *aroG,* determines DAHP synthase, the first enzyme of the aromatic amino acid biosynthetic pathway, converting erythro-4-phosphate and phosphoenolpyruvate to 3-deoxy-D-arabino-heptuosonate-7-phosphate. Notably, the *aroG* gene has been identified in a recent in vivo signature-tagged mutagenesis (STM) approach of LVS [1]. However, its in vitro properties were not characterized.

One mutant had an insertion into gene *FTL_0116* (or *FTL_1162*). *FTL_0116* corresponds to the gene *pdpC* of the Francisella pathogenicity island (FPI). Of note, attenuated mutants with a single insertion in several genes of one of the two copies of the duplicated FPI locus of LVS have also been identified in others screens [1,2], suggesting that the copy number of the FPI members may be important for virulence. One insertion mutant was isolated in gene *FTL_1724*, encoding a hypothetical protein of unknown function. One insertion fell into gene *FTL_1116*, encoding a putative ATP-dependent DNA helicase of the UvrD/RepA family. Finally, three insertions occurred into intergenic regions (upstream of *FTL_1009, FTL_1037* and *FTL_1519*, respectively), one into a transposase gene (*FTL_0861*) and one into a pseudogene (*FTL_0096*). A double-insertion mutant that carried two insertions was also sequenced: it carried one insertion into gene *FTL_0148*, encoding a putative sodium/hydrogen exchanger and a second insertion in an intergenic region.

**Intracellular growth and virulence of the *F. tularensis* mutants.** The kinetic of intracellular multiplication of the mutants was individually followed in J774 cells, over a 48h-period. Three categories of intracellular growth defects were observed.

Mutant *FTL_0766* (*ggt*) had a drastic growth defect, with more than a 100-fold reduction of intracellular growth.

Mutant *FTL_1240* (*aroG*) showed a ≥10-fold reduction of intracellular growth. We found here that in both J774 cells and in BMM, the *aroG* mutation reduced only moderately bacterial multiplication and persistence. Six mutants showed only a mild (≤10-fold reduction of multiplication, after 24h (3 mutants) or 48h (3 mutants). Finally, 3 mutants (*FTL_0096; FTL_1037* and *FTL_1519*, respectively) did not show any growth defect as compared to LVS; suggesting that their growth defect might be visible only when present in pools of mutants. For phenotypes of mutants see SI Table 1.

The impact of gene inactivation on bacterial virulence was also evaluated for 6 mutant strains by infecting 6-8 weeks old BALB/c mice by the intraperitoneal (i.p.) route with serial ten fold dilutions of bacteria. The survival of the mice was followed for 9 days. The median lethal dose was determined to be 10^1.04^ for LVS; 10^1.6^ for *FTL_0148*; 10^1.1^ for *FTL_1116*; 10^2.6^ for *FTL_0116* (or *FTL_1162*); 10^3.1^ for *FTL_1724*; 10^4.8^ for *FTL_0766* (*ggt*); and 10^7.2^ for *FT_1240* (*aroG*) using the Probit method [3].

**Supplementary Materials and Methods.**

**DNA manipulations.** Plasmid DNA and *Francisella* genomic DNA was purified using the QIAprep Spin Miniprep Kit and DNeasy Blood & tissue Kit, respectively (Qiagen, Valencia, CA). DNA fragments were purified using QIAquick gel extraction kit (QIAGEN). Restriction enzyme digestions and ligations were performed as recommended by the manufacturer (New England Biolabs). PCR was done using DyNAzymeII DNA polymerase (Finnzymes, Espoo, Finland) on an iCycler (Biorad, Marne La Coquette).

**Cell cultures and macrophage infections.** One day before the start of an experiment, J774 cells were detached, enumerated and diluted in culture medium, Dulbecco’s modified Eagle medium (DMEM, PAA Laboratories GmbH, Pasching) with 10% *(vlv)* fetal calf serum to final concentration of 2x10^5^ cells ml^-1^. One ml of cell suspension was distributed in each well of a 24-well tissue culture plate. After incubation overnight at 37°C and 5% CO_2_, wells were washed and reconstituted with fresh culture medium with 5% *(vlv)* fetal calf serum containing 2x10^7^ bacteria ml^-1^. Bacterial uptake was allowed to occur for a 1-h period. After bacterial uptake, the monolayers were washed three times with fresh culture medium and incubated for indicated periods of time in culture medium with 5% *(vlv)* fetal calf serum and 10 µg m1^-1^ of gentamycin.

**Southern blot analysis.** For each Km^R^ colony recovered after in vitro negative selection in J774 cells, *HimarFT* transposition was verified by Southern blot analysis. Genomic DNA (5 µg) was digested with *Spe*I overnight, resolved on a 0.7% TAE-agarose gel and transferred to a positively charged nylon membrane (Hybond N+, Amersham Pharmacia) by capillary action for 4h. Randomly labeled DNA fragments (prepared using Rediprime II, Amersham Pharmacia) were used as probes. Two different probes were prepared: i) a 634-bp fragment corresponding to the neomycin phosphotransferase (*npt*) gene; and ii) a 624-bp fragment corresponding to the β-lactamase (*bla*) gene. Labeling and detection were performed as recommended by the manufacturer.

**Determination of the insertion site.** *HimarFT* insertion sites were mapped, as described previously [4]. Genomic DNA was isolated from each mutant strain and 1μg was digested with *Spe*I overnight. The enzyme was inactivated at 65°C for 20 min and digested genomic DNA was ligated with T4 DNA ligase. *Himar*-containing fragments were recovered as plasmids in *E. coli* DH5α λpir. The *HimarFT* insertion site was determined by sequencing and comparison with the *F. tularensis* LVS genome. DNA sequencing was performed by CHU Cochin, Paris.

**Electroporation.** Electroporation of electrocompetent *E.coli* DH5α λpir bacteria was performed by using a Bio-Rad Gene Pulser (2.5 kV, 25µF, 200Ω). After electroporation, cells were immediately added to 1 ml of LB medium, incubated with shaking for 1h at 37°C and plated on LB agar with the appropriate antibiotic. For electroporation of *F. tularensis* LVS, cultures (50 ml) were grown to the mid-exponential phase in Schaedler K3 (OD_600_, 0.3-0.6), washed twice with 0.5 M sucrose and suspended in ~1 ml of 0.5 M sucrose to obtain a concentration of ~ 1×10^10^ cells ml^-1^. For electroporation, ~1μl of plasimd DNA (~100 µg/ml) was mixed with 200µl of electrocompetent cells, incubated at room temperature for two min, and electroporated in a 0.2cm cuvette (2.5 kV, 25 µF, 600 Ω). Immediately after electroporation, cells were suspended in 1 ml of Schaedler K3 and incubated at 30°C for 5h before selection on Schaedler agar containing the appropriate antibiotic.

**Sensitivity to human serum.** The sensitivity assay was performed essentially as described previously [5]. Briefly, dilutions of 10^6^ bacteria in RPMI-Hepes (20 mM) of LVS, *wbtA* and *ggt* mutants were incubated at 37°C for 1h in the presence of increasing concentration of non-decomplemented human serum (0-20%) (PAA Laboratories, GmbH Pasching). Serial dilutions in RPMI-Hepes were plated on chocolate agar plates to determine the number of viable bacteria at the beginning and end of the experiment.

**References**

1. Su J, Yang J, Zhao D, Kawula TH, Banas JA, et al. (2007) Genome-wide identification of *Francisella tularensis* virulence determinants. Infect Immun 75: 3089-3101.

2. Maier TM, Casey MS, Becker RH, Dorsey CW, Glass EM, et al. (2007) Identification of *Francisella tularensis* Himar1-based transposon mutants defective for replication in macrophages. Infect Immun 75: 5376-5389.

3. Roth Z (1961) [A graphic probit method for the calculation of LD50 and relative toxicity.]. Cesk Fysiol 10: 408-422.

4. Maier TM, Pechous R, Casey M, Zahrt TC, Frank DW (2006) In vivo Himar1-based transposon mutagenesis of *Francisella tularensis*. Appl Environ Microbiol 72: 1878-1885.

5. Sandstrom G, Lofgren S, Tarnvik A (1988) A capsule-deficient mutant of *Francisella tularensis* LVS exhibits enhanced sensitivity to killing by serum but diminished sensitivity to killing by polymorphonuclear leukocytes. Infect Immun 56: 1194-1202.
